# Molecular dynamics simulation in traditional Chinese medicine research: from molecular mechanisms to multiscale validation

**DOI:** 10.3389/fchem.2026.1879034

**Published:** 2026-07-23

**Authors:** Xinyi Zhou, Haitao Du, Haotian Yang, Yanan Hu, Cheng Wang, Mengru Zhang, Xiaoyan Ding, Ping Wang

**Affiliations:** 1 School of Pharmacy, Shandong University of Traditional Chinese Medicine, Jinan, China; 2 Shandong Academy of Chinese Medicine, Jinan, China

**Keywords:** bioactive compound screening, high-throughput screening, molecular dynamics simulation, target mechanisms, traditional Chinese medicine drug development

## Abstract

Molecular dynamics (MD) simulation is a computational technique based on Newtonian mechanics and statistical physics that tracks molecular motion and ligand-target recognition at atomic resolution. In recent years, with advances in computational resources and continuous optimization of simulation algorithms, the application of MD simulations in traditional Chinese medicine (TCM) research and development has expanded steadily. Because TCM involves chemically diverse constituents, multiple targets, metabolic transformation, and pathway-level regulation, its mechanisms are difficult to interpret using static or single-target approaches alone. Owing to its high spatiotemporal resolution, MD simulation can characterize binding modes, structural adaptation, and interaction patterns of representative TCM constituents with target proteins. When combined with network pharmacology, multi-omics, artificial intelligence (AI)-assisted modeling, and experimental validation, MD-derived evidence can help transform static compound–target associations into testable mechanistic hypotheses. This review summarizes the methodological development, software platforms, databases, and computational workflows of MD simulation, with emphasis on their relevance to TCM-oriented mechanism research. Representative applications involving flavonoids, alkaloids, glycosides, terpenoids, and polysaccharides are discussed to show how MD provides molecular-level evidence for selected compound-target interactions, membrane-associated behavior, receptor recognition, and free-energy profiles. The review also highlights key challenges, including high computational cost, insufficient sampling, uncertain force-field parameters, difficulty in modeling multicomponent systems, limited trajectory reproducibility, and insufficient experimental validation. Looking ahead, the further integration of artificial intelligence, network pharmacology, and high-throughput screening is exp ected to enhance the role of MD simulations in the modernization of TCM research.

## 1 Introduction

Molecular dynamics (MD) simulation is a computational method grounded in Newtonian mechanics and statistical mechanics. By numerically integrating the equations of motion, it simulates and predicts the dynamic behavior of molecular systems under defined physical conditions (Farhadi et al., 2025; Nencini et al., 2024). At atomic resolution, MD can capture the time evolution of molecular structures, thereby compensating for the limited spatiotemporal resolution of conventional experimental approaches (Persson et al., 2024; Zadorozhnyi et al., 2024). It has been widely used in biology, chemistry, materials science, drug design, and related fields, and has become an important tool for linking microscopic atomic motions with macroscopic physicochemical phenomena, providing a dynamic framework for mechanistic investigation and functional design (Kotschy et al., 2024).

Traditional Chinese medicine (TCM) refers to natural medicines used for the prevention, diagnosis, and treatment of disease under the guidance of traditional Chinese medical theory, particularly the principles of holism and syndrome differentiation (Hua et al., 2025). A defining feature of TCM is its multicomponent, multitarget, and multipathway mode of action, which gives rise to complex systemic regulation and dynamic synergistic effects (Chen B. et al., 2025; Li C. et al., 2025). In recent years, with the rapid development of artificial intelligence (AI), big-data analytics, and multi-omics technologies, research on TCM modernization has gradually shifted from empirical interpretation toward a more data-driven paradigm (Song et al., 2024). In this review, AI mainly refers to computational approaches that support protein structure prediction, compound and target prioritization, adaptive sampling, machine-learning-based force fields, and molecular design. Network pharmacology, through the construction of “compound-target-pathway” networks, has provided a systems-level framework for understanding the multicomponent and multitarget mechanisms of TCM (Huang et al., 2024; Chen et al., 2025a). Multi-omics approaches have further revealed the global biological responses induced by TCM interventions at the level of expression regulation (Sun et al., 2024). Molecular docking, as a structure-based virtual screening approach, has also played an important role in the preliminary screening of active compounds and the prediction of potential targets (Chen et al., 2025). Meanwhile, AI-assisted structure prediction and drug-screening algorithms have markedly improved the efficiency of candidate identification and broadened the scope of accessible targets (Abramso et al., 2024; Zhou et al., 2025).

However, most of these methods rely primarily on static structural matching or correlation-based analysis, making them less capable of describing protein conformational dynamics and energy transfer under physiological conditions (Roy R. K. et al., 2025). Because of the chemical complexity of TCM and its multilayered mechanisms of action, its active material basis and relevant targets often remain insufficiently defined, and robust, quantitative, and reproducible research strategies are still lacking (Tran et al., 2025). This has become one of the major bottlenecks in the modernization of TCM. By resolving molecular motion at atomic resolution, MD simulation helps overcome some limitations of conventional structure-function analysis (Shekhar et al., 2022). In TCM research, its value is particularly evident. By capturing time-dependent molecular interactions at the atomic level, MD can clarify the binding modes, conformational adaptation, and key residue interactions of representative TCM constituents with specific protein targets (Liang et al., 2024; Yi et al., 2025). These results provide dynamic molecular evidence for selected compound-target relationships and can be integrated with network pharmacology and multi-omics data to support the broader interpretation of multicomponent and multitarget mechanisms in TCM (Yang et al., 2025). Compared with approaches based mainly on static structures, MD provides additional dynamic evidence for identifying core active constituents, evaluating predicted target interactions, and generating mechanistic hypotheses related to TCM formula compatibility (Gu et al., 2024; Ye et al., 2024).

In TCM research, computational analysis often begins with the identification of candidate constituents, targets, and pathways from databases, network pharmacology, and multi-omics data (Qin et al., 2025; Ren et al., 2025). These approaches are useful for narrowing the search space, but they usually provide static or correlation-based associations rather than direct evidence of molecular behavior (Buttenschoen et al., 2024). MD simulation can add a dynamic layer to this workflow by evaluating whether predicted compound-target interactions remain stable over time, whether ligand binding induces local or global conformational adaptation, and whether membrane environments, solvent effects, or multiple constituents influence target recognition (Cui et al., 2024; Ma et al., 2025). In this sense, MD does not simply serve as a final confirmation step after docking; it helps transform predicted associations into mechanistic hypotheses that can be tested experimentally.

Against this background, this review systematically discusses the current development of MD simulation, its core methods and platforms, and its specific applications in TCM drug development. It further analyzes the major challenges currently facing this field and considers future directions, with the aim of providing a useful reference for the broader application, innovation, and continued development of MD-based approaches in TCM research.

## 2 Development and advantages of molecular dynamics simulation

**FIGURE 1:**
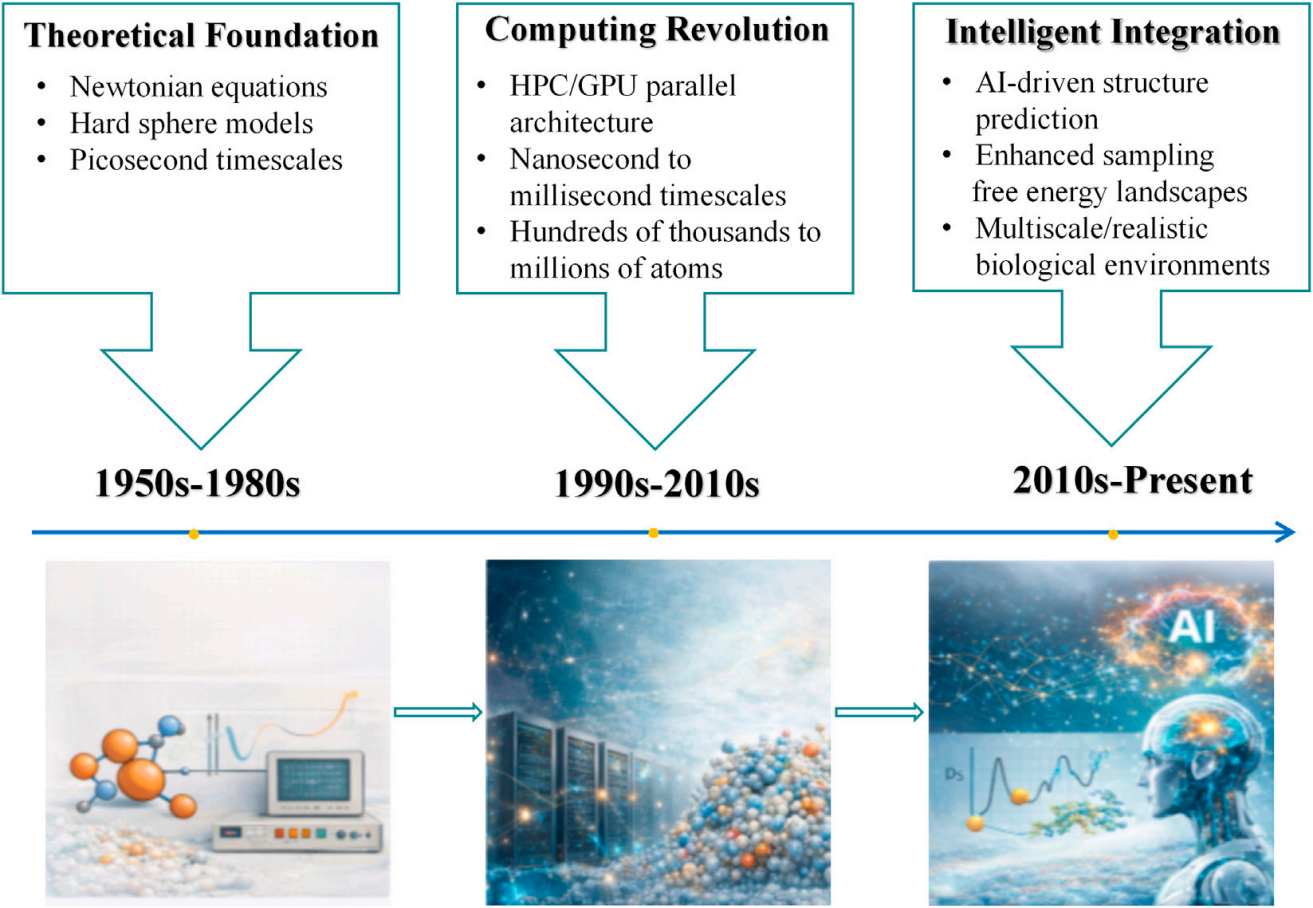
Historical timeline of molecular dynamics simulation development and application expansion.

Since its emergence in the 1950s, molecular dynamics simulation has undergone three broad stages of development: the stage of theoretical foundation, the stage of computational expansion, and the stage of intelligent integration (Figure 1).

### 2.1 Development of MD simulation

#### 2.1.1 Theoretical foundation: early development in computational physics

The MD method is generally considered to have originated from the event-driven simulation of hard-sphere systems conducted by Alder and Wainwright in 1957. At this early stage, simulations were primarily applied to idealized particle models and simple fluid systems. Owing to the severe limitations of contemporary computing resources, the simulated systems were small and the accessible trajectories were extremely short. Nevertheless, these pioneering studies demonstrated that the time evolution of interacting many-particle systems could be treated computationally, thereby establishing an important conceptual foundation for molecular simulation (Hollingsworth and Dror, 2018). In 1977, McCammon, Gelin, and Karplus reported one of the earliest atomistic molecular dynamics simulations of a biological macromolecule, using bovine pancreatic trypsin inhibitor as a model protein (McCammon et al., 1977). This study extended MD from idealized physical systems to biological macromolecules and demonstrated that proteins should be regarded not as rigid structures, but as dynamic molecular systems undergoing continuous atomic fluctuations. It also provided an important conceptual and technical foundation for subsequent investigations of protein conformational dynamics, folding, molecular recognition and ligand binding (Karplus and McCammon, 2002).

Early biomolecular MD simulations, however, remained strongly constrained by computational cost. Simulated systems generally contained only hundreds to a few thousand atoms, while trajectory lengths were commonly restricted to the picosecond range. For example, the original bovine pancreatic trypsin inhibitor (BPTI) simulation generated a trajectory of approximately 9.2 ps. In addition, the empirical potential functions and parameter sets available at the time provided relatively simplified descriptions of biomolecular interactions. Together with the short trajectory lengths, these limitations resulted in restricted conformational sampling and limited predictive reliability. Consequently, early biomolecular MD functioned primarily as an exploratory and proof-of-concept approach rather than as a quantitatively mature predictive method (Adcock et al., 2006).

#### 2.1.2 Computational expansion: scale transition driven by high-performance computing

From the 1990s onward, with the rapid development of high-performance computing architectures, molecular dynamics simulation entered a stage of large-scale expansion driven primarily by computational power (Phillips et al., 2005). The establishment of parallel computing frameworks made it possible to partition and compute large atomic systems, while the introduction of graphics processing unit (GPU) accelerators and distributed computing platforms further improved trajectory sampling efficiency per unit time (Friedrichs et al., 2009). During this period, simulations began to cover systems containing hundreds of thousands or even millions of atoms (Sikora et al., 2021). The accessible timescale also extended from nanoseconds to microseconds, and in some cases to milliseconds (Lindorff-Larsen et al., 2011). At the same time, classical force fields such as AMBER (Case et al., 2005), Chemistry at Harvard Macromolecular Mechanics (CHARMM) (Huang et al., 2017), and optimized potentials for liquid simulations (OPLS) (Roos et al., 2019) were continuously refined, improving the accuracy of protein conformational stability, secondary-structure retention, and solvation effects. Enhanced-sampling methods such as Replica Exchange MD (Stratmann et al., 2025), accelerated molecular dynamics (Miao and McCammon, 2017), and metadynamics (Invernizzi et al., 2020) enabled the crossing of high free-energy barriers and enabled more efficient exploration of conformational ensembles and free-energy landscapes. Using all-atom simulations, Freddolino et al. (2006) successfully revealed the dynamic interactions between viral capsid proteins and RNA, representing a landmark achievement of this stage.

However, despite these advances, conventional all-atom MD remains limited when applied to very large systems, long-timescale conformational events, and complex multicomponent environments. In this context, coarse-grained molecular dynamics (CG-MD) was further developed and increasingly adopted as an important multiscale strategy for overcoming the spatial and temporal limitations of atomistic simulations. By mapping groups of atoms into simplified interaction sites, CG-MD substantially reduces the number of degrees of freedom and allows larger systems and longer timescales to be explored (Borges-Araújo et al., 2023; Ugarte La Torre and Sugita, 2025). Although this reduction in resolution may limit the description of specific hydrogen-bond networks and residue-level interactions, CG-MD is particularly valuable for studying large biomolecular assemblies, membrane environments, molecular aggregation, and multicomponent systems (Lindsay and Li, 2024; Ingólfsson et al., 2023). These features are highly relevant to TCM research, where formulae often contain multiple structurally diverse constituents that may coexist in membrane or protein-rich environments and act through weak, cooperative, or competitive interactions (Boopathi and Garduño-Juárez, 2024; Ji et al., 2024). Therefore, CG-MD and all-atom MD should be regarded as complementary approaches: CG-MD can be used to identify long-timescale distribution patterns, aggregation behavior, and global conformational changes, whereas all-atom MD can further refine local binding modes and key residue interactions.

#### 2.1.3 Intelligent integration: the era of informatization and artificial intelligence

Since the 2010s, the focus of MD development has gradually shifted toward a stage of intelligent integration centered on data-driven algorithm optimization and model reconstruction, and progress in this period has relied more heavily on data-driven methods and the coordinated integration of multiple technologies (Zhang J. et al., 2023). In combination with MD, especially in the study of complex systems such as membrane proteins and drug targets, deep-learning-based protein structure prediction tools represented by AlphaFold have provided more accurate initial conformations, thereby improving both simulation efficiency and accuracy. For example, in studies of the binding mechanisms between flavonoid compounds and cancer-related proteins, target protein structures predicted by AlphaFold and subsequently combined with MD simulations have successfully revealed key hydrogen-bonding and hydrophobic interactions between flavonoid molecules and protein surfaces, providing a theoretical basis for new targeted drug design (Jumper et al., 2021). Meanwhile, the combination of enhanced-sampling algorithms with machine learning, through the construction of low-dimensional reaction coordinates or the use of neural networks to identify key dynamical variables, has enabled more accurate descriptions of free-energy landscapes and improved the analysis of conformational transition pathways and steady-state distributions (Zhu et al., 2026). The scope of MD has also expanded from simple protein-small-molecule systems to multiscale coupled systems. By integrating all-atom simulations, coarse-grained models, and continuum models, it has become possible to connect different temporal and spatial scales and achieve cross-scale modeling from the molecular level to the cellular environment (Iwata, 2024). At present, MD simulation has evolved from a theoretical simulation tool into a core technology in new-drug development, materials design, and protein structure-function analysis.

In practical TCM-oriented workflows, AI-assisted MD can operate at several stages. Protein structure prediction tools can provide initial models for targets without experimentally resolved structures, although these models still require refinement because predicted conformations may not represent ligand-bound, allosteric, or membrane-associated states (Dobson et al., 2023; Raisinghani et al., 2024). Machine-learning-based potentials, such as deep potential models implemented in DeePMD-kit, may improve the balance between accuracy and efficiency when modeling structurally diverse natural products (Zeng et al., 2023). Adaptive sampling approaches can analyze preliminary trajectories, identify under-sampled conformational regions, and guide additional simulations. For example, DeepDriveMD uses deep learning to construct latent representations from MD trajectories and steer subsequent sampling (Gupta et al., 2024). In addition, generative molecular design can be combined with MD-derived binding modes, key residue interactions, and free-energy profiles to optimize TCM-derived scaffolds or analogues (Loeffler et al., 2024). Together, these approaches make AI a workflow-level complement to MD rather than a substitute for physical simulation.

### 2.2 Advantages of molecular dynamics simulation

A major advantage of MD simulation is its ability to generate atomically resolved, time-dependent conformational ensembles, thereby complementing the discrete structural snapshots obtained from X-ray crystallography and cryo-electron microscopy. By sampling protein and ligand motions, MD can characterize conformational rearrangements, induced-fit and conformational-selection processes, conformational transition pathways, metastable intermediates, and transient or cryptic allosteric pockets that may be difficult to detect experimentally. Recent studies have prospectively predicted allosteric fragment-binding sites and binding poses using long-timescale MD and subsequently validated these predictions by X-ray crystallography (Greisman et al., 2023). Enhanced-sampling MD combined with functional experiments has also revealed cryptic-pocket opening and ligand recognition (Haloi et al., 2025), while computational reanalysis of crystallographic data has uncovered low-occupancy ligand-bound states and previously unrecognized allosteric sites that may be missed by conventional structural analysis (Mehlman et al., 2024). Together, these findings illustrate how dynamic simulations and complementary computational approaches can uncover pharmacologically relevant conformational states that are poorly represented in conventional structural datasets.

KRAS illustrates how identifying previously unrecognized binding pockets can transform a difficult target into a druggable one. In 2013, covalent-fragment screening and crystallographic analysis revealed the switch-II pocket in guanosine diphosphate (GDP)-bound KRAS G12C, enabling mutant-selective inhibition (Ostrem et al., 2013). Subsequent studies showed that these inhibitors covalently bind KRAS G12C and stabilize its inactive GDP-bound state (Lito et al., 2016). Structure-guided optimization led to the development of sotorasib (Canon et al., 2019; Lanman et al., 2020), whose clinical efficacy was demonstrated in phase I and II trials (Hong et al., 2020; Skoulidis et al., 2021) and ultimately supported its U.S. Food and Drug Administration (FDA) approval as the first targeted therapy for KRAS G12C-mutated non-small-cell lung cancer (Nakajima et al., 2022). This example highlights the value of integrating computational, structural, biochemical, and pharmacological approaches to identify druggable conformational states and guide rational drug development.

## 3 Software and algorithms related to molecular dynamics simulation

**FIGURE 2:**
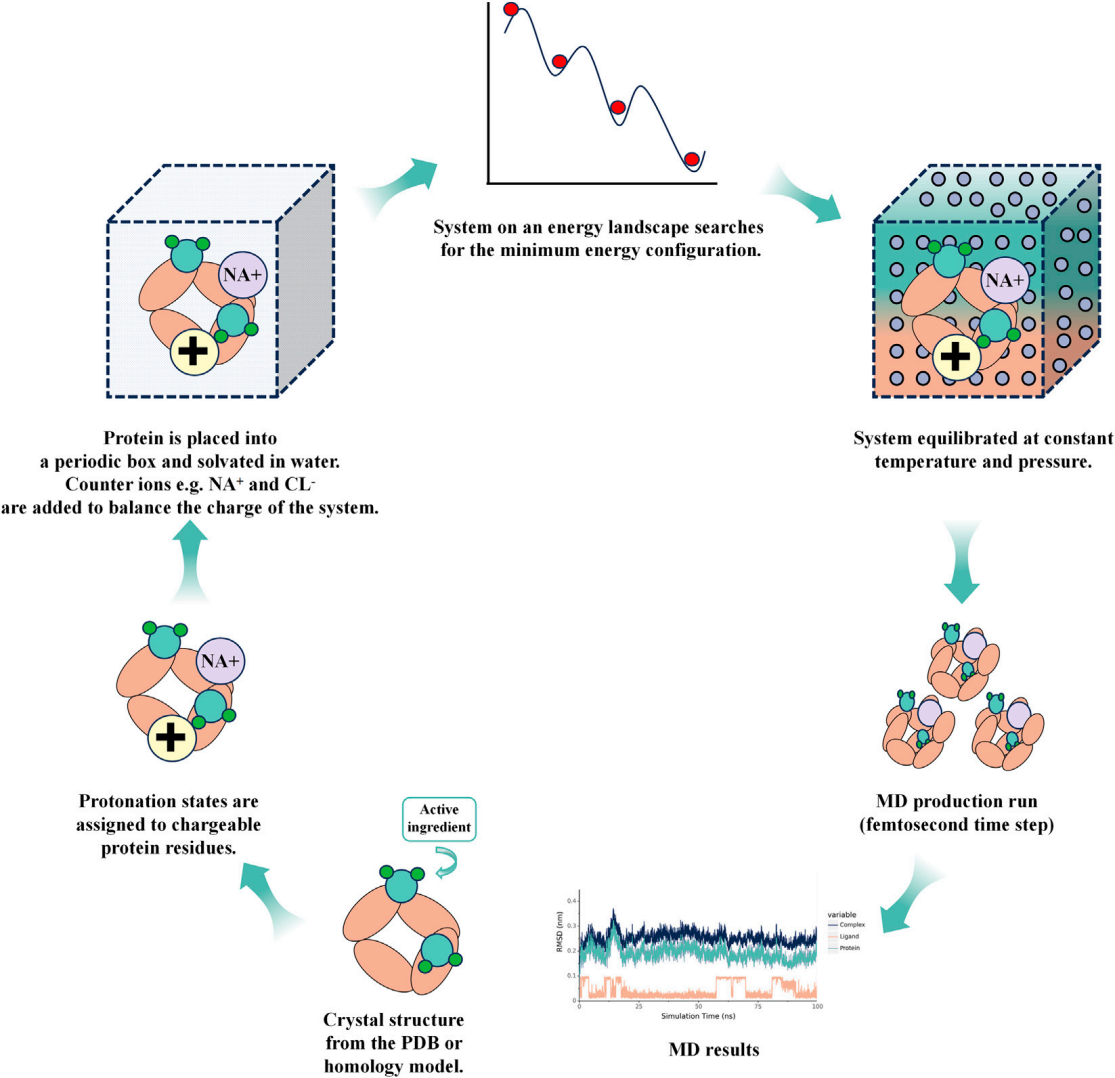
Typical all-atom molecular dynamics simulation workflow diagram.

The MD workflow can be divided into four key steps: system construction, energy minimization and equilibration, production simulation, and trajectory analysis (Lemkul, 2024) (Figure 2). First, during system construction, the protein structure is prepared, the ligand is parameterized, and the solvent model and ions are added to ensure that the simulation system is physically reasonable under the selected force-field parameters. This is followed by energy minimization and equilibration, to remove structural artifacts and stabilize the system at the target temperature and pressure. On this basis, the system then enters the production phase, in which integration algorithms are used to propagate the system and generate trajectory data that reflect atomic motion (Rizzuti, 2022). This stage is the core step for obtaining dynamic information such as protein-ligand interactions and conformational changes, and typically uses femtosecond time steps with total simulation times ranging from nanoseconds to microseconds (Sahil et al., 2023). Finally, trajectory analysis is performed to extract key information, such as root-mean-square deviation (RMSD), root-mean-square fluctuation (RMSF), hydrogen-bond networks (Sinha et al., 2022), and free energy (Valdés-Tresanco et al., 2021), in order to evaluate binding stability and conformational adaptability and to reveal possible mechanisms of action. Because the simulation process involves the calculation of motions for a large number of atoms, it requires substantial computational resources and therefore depends on high-performance computing clusters and suitable software support (Páll et al., 2020). At the same time, the force-field models (Robustelli et al., 2018), integration algorithms (Shirts et al., 2017), and enhanced-sampling methods (Bernardi et al., 2015) adopted by different software platforms directly determine the accuracy and efficiency of the simulation. Table 1 summarizes representative MD packages and related computational tools, with emphasis on simulation scale, GPU support, typical TCM-MD applications, and compatibility with commonly used workflows.

**TABLE 1:** Representative MD simulation packages and computational tools for TCM-oriented molecular modeling.

Category	Software/Methods	Abstract	GPU support	Typical applications in TCM-MD research	Compatibility	Advantage	Disadvantage
Core MD simulation package	GROMACS (Pronk et al., 2013)	An open-source and high-performance MD simulation package widely used for biomolecular systems, including proteins, protein-ligand complexes, nucleic acids, lipid membranes, and coarse-grained systems. It is suitable for all-atom and CG-MD simulations in TCM-related target and membrane studies	Yes	Binding stability analysis of TCM constituents, membrane simulations, CG-MD with Martini, batch simulations of representative compound-target pairs	Compatible with PLUMED, Martini, CHARMM-GUI, ACPYPE, gmx_MMPBSA	Highly efficient, open-source, and widely adopted. It supports multiple force fields, GPU acceleration, large-scale simulations, and extensive trajectory analysis. It can also be integrated with PLUMED for enhanced sampling	System setup and parameter preparation require technical expertise. For structurally complex TCM constituents, additional ligand parameterization and careful validation are often needed
Core MD simulation package	AMBER (Case et al., 2025)	A mature biomolecular MD simulation suite widely used for proteins, nucleic acids, protein-ligand complexes, and binding free-energy analysis	Yes, mainly through pmemd.cuda	TCM-derived ligand-target simulations, free-energy analysis, residue decomposition	Compatible with AmberTools, Antechamber, MMPBSA.py, ACPYPE	Provides a well-established force-field ecosystem and strong support for ligand parameterization, trajectory analysis, and molecular mechanics generalized Born surface area (MM/GBSA) or MM/PBSA calculations	Some versions require licensing. Complex natural products may require careful parameter validation
Core MD simulation package	OpenMM (Eastman et al., 2017)	A Python-friendly MD platform suitable for batch simulation of TCM constituents and customized force-field testing. It supports rapid prototyping and is appropriate for preliminary screening of candidate molecules over 10–50 ns	Yes	Automated workflows, AI-assisted MD, high-throughput simulation of TCM candidates	Python-based; compatible with multiple force-field formats and customized workflows	Well suited for rapid prototyping and batch processing. Its Python interface facilitates integration with other tools and datasets, and its efficient simulation performance makes it useful for TCM molecular screening	Relatively limited for long-timescale simulations and requires a certain level of familiarity with Python programming
Core MD simulation package	NAMD (Phillips et al., 2020)	Suitable for large-scale systems and long-timescale MD simulations, especially for multimeric proteins or transmembrane proteins. When combined with enhanced-sampling techniques (REST2/GaMD), it can be used to evaluate the binding stability of proteins and drugs	Yes	Large protein complexes, membrane-associated targets, long-timescale simulations	Compatible with CHARMM force fields, VMD, PLUMED	Appropriate for complex and large-scale systems, supports enhanced-sampling strategies, and can improve simulation accuracy	Has relatively high hardware requirements, consumes substantial computational resources, and requires considerable expertise for operation and debugging
Enhanced sampling tool	PLUMED 2 (Gioia et al., 2017)	A tool for metadynamics and umbrella sampling, often used together with other simulation platforms (such as GROMACS and NAMD) to analyze the binding process of TCM constituents	Depends on linked MD engine	Metadynamics, umbrella sampling, free-energy landscape analysis of TCM ligand binding	Compatible with GROMACS, NAMD, OpenMM and other MD engines	Specialized for barrier crossing and free-energy calculations, highly flexible, and suitable for complex drug molecules	Must be used in combination with other MD platforms
Core MD simulation package	Desmond (Alghamdi et al., 2023)	A commercial high-speed MD platform that uses the OPLS force field and enhanced-sampling techniques. It is suitable for simulations involving flexible binding pockets and can analyze the stability of drug-target interactions over long simulations of 200–500 ns	Yes	Long-timescale ligand-target stability analysis and workflow-based TCM compound screening	Integrated with Schrödinger workflows and OPLS force fields	Fast and efficient, suitable for large-scale and long-timescale simulations	Requires a paid license, is not open-source, and has relatively high hardware demands
Binding free-energy analysis	MMPBSA.py (Miller et al., 2012)	A tool for estimating binding free energy from MD trajectories. It can perform approximate calculations using the MM/GB(PB)SA approach and provides energy decomposition analysis to identify key binding sites	Not primarily GPU-dependent	MM/PBSA or MM/GBSA estimation, residue-level energy decomposition for TCM ligand–target complexes	AMBER and GROMACS-compatible workflows	Provides energy decomposition analysis and supports binding free-energy calculations through the MM/GB (PB) SA method	If the simulation time is too short, the accuracy of the estimated binding free energy may be affected
Structure preparation and parameter conversion	ACPYPE (Kagami et al., 2023)	Used to convert small-molecule parameters generated by Antechamber into formats compatible with GROMACS and related platforms, thereby ensuring data compatibility and conversion efficiency across different software environments	Not primarily GPU-dependent	Parameter preparation for TCM-derived small molecules and conversion between AMBER and GROMACS formats	AMBER and GROMACS workflows	Ensures compatibility among different software platforms and improves the efficiency of format conversion	Support for certain specific formats is limited, and not all software is fully compatible
Multiscale simulation approach	CG-MD based on the Martini force field (Singh and Li, 2019)	A multiscale MD strategy that maps groups of atoms into simplified interaction sites, allowing larger systems and longer simulation timescales. It is suitable for modeling membrane systems, molecular aggregation, large biomolecular assemblies, and multicomponent TCM systems	Depends on MD engine, commonly supported through GROMACS	Multicomponent coexistence, membrane distribution, aggregation, polysaccharides, saponins, volatile-oil systems	Commonly used with GROMACS; compatible with Martini ecosystem	Significantly reduces computational cost and expands accessible spatial and temporal scales. It is useful for studying membrane distribution, aggregation behavior, and cooperative interactions among multiple TCM constituents	Lower structural resolution than all-atom MD; limited ability to describe specific hydrogen bonds, fine residue-level interactions, and detailed ligand recognition. Parameterization of structurally complex TCM natural products remains challenging
System construction	CHARMM-GUI (Jo et al., 2017)	A web-based platform for preparing protein, membrane, carbohydrate, glycoprotein, and protein-ligand simulation systems	Not applicable	Preparing membrane proteins, glycosides, polysaccharides, and complex biomolecular systems for TCM-MD	Generates inputs for GROMACS, AMBER, NAMD, CHARMM, OpenMM	Particularly useful for constructing complex membrane and biomolecular systems, and can generate input files for multiple MD packages	Uncommon TCM natural products may still require external parameterization and manual inspection

## 4 Major databases, repositories, and tools supporting MD-based TCM research

The depth and reliability of MD studies depend to a large extent on the accuracy and completeness of the initial structures, force-field parameters, and associated analytical tools used in the simulation. To improve standardization and research efficiency, a series of data repositories and integrated simulation platforms have been developed to meet simulation needs at different levels and for different purposes. Table 2 summarizes major databases, repositories, and auxiliary tools that support MD-based TCM research, including their accessibility, typical uses, strengths, and limitations.

**TABLE 2:** Overview of major functional databases and tools for molecular dynamics simulation.

Category	Databases/Tools	Main content	Typical use in TCM-MD research	Strengths	Limitations
MD trajectory repositories	NMRlipids Databank (Kiirikki et al., 2024)	Lipid membrane MD trajectories and lipid force-field validation data	Reference for membrane-environment simulations involving saponins, terpenoids, volatile oils, or membrane proteins	Useful for membrane model validation and lipid parameter comparison	Mainly focused on lipid systems; limited TCM-specific trajectories
	MDRepo (Roy A. et al., 2025)	Community-generated biomolecular MD trajectories	Trajectory sharing, reuse, benchmarking, and reproducibility analysis	Supports open MD data deposition and reuse	TCM-specific systems are still limited
	MDverse (Tiemann et al., 2024)	Indexed MD datasets and trajectory resources	Improving findability and reuse of MD datasets	Supports FAIR-oriented discovery of MD data	Coverage and metadata consistency vary across datasets
TCM constituent/formula databases	HERB (Fang et al., 2021)	Herbs, constituents, targets, diseases, and supporting evidence	Candidate constituent and target selection before docking and MD	Integrates literature and high-throughput data	Target annotations require cross-validation
	ETCM (Xu et al., 2019)	Formulae, herbs, ingredients, targets, and phenotype annotations	Formula-level compound and target screening	Strong formula and phenotype coverage	Predicted targets need experimental confirmation
	TCMID 2.0 (Huang et al., 2018)	TCM formulae, herbs, ingredients, targets, and related annotations	Cross-checking TCM constituents, synonyms, and target information	Broad coverage of TCM-related entities	Data redundancy and identifier inconsistency may require manual curation
	SymMap (Wu et al., 2019)	Symptom, target, disease, herb, and ingredient mapping	Building syndrome-target-pathway relationships for target prioritization	Links TCM symptoms with modern biomedical concepts	Mechanistic links are partly inferred and need validation
	DCABM-TCM (Liu et al., 2023)	Blood-absorbed constituents and metabolites of TCM	Prioritizing *in vivo* relevant compounds for MD simulation	More consistent with absorbed pharmacodynamic substances	Coverage remains limited compared with general compound databases
	TCMSP (Ru et al., 2014)	TCM ingredients, ADME-related parameters, and predicted targets	Preliminary ADME-based screening and network construction	Widely used in TCM network pharmacology	OB/DL filters may exclude active metabolites or low-abundance constituents
Protein structure and annotation databases	RCSB PDB (Burley et al., 2023)	Experimentally determined macromolecular structures	Obtaining target structures for docking and MD	High-quality experimental structures	Some TCM-related targets lack resolved structures or ligand-bound conformations
	UniProt (UniProt Consortium, 2023)	Protein sequences, isoforms, functions, and annotations	Target curation, species confirmation, and functional annotation	Standardized protein information	Does not provide MD-ready simulation systems
	AlphaFold DB (Varadi et al., 2022)	Predicted protein structures at proteome scale	Initial structure modeling when experimental structures are unavailable	Broad target coverage	Predicted structures may not represent active, ligand-bound, or membrane-associated states
	PDBbind (Wang et al., 2004)	Protein-ligand complexes with binding affinity data	Benchmarking docking and free-energy calculations	Useful for method calibration and affinity reference	Limited coverage of TCM-derived natural products
Molecular visualization tools Network and visualization tools Pharmacophore and screening tools Chemical identifiers and structure tools	Mol* Viewer (Sehnal et al., 2021)/3Dmol.js (Rego and Koes, 2015)	Interactive molecular structure and trajectory visualization	Displaying protein structures and MD-related results	Lightweight and useful for reporting and sharing results	Limited advanced trajectory analysis compared with dedicated MD software
	Cytoscape string APP (Doncheva et al., 2019)	PPI network integration and visualization with STRING/STITCH	Identifying key network nodes for docking and MD validation	Strong network visualization and integration	Not an MD simulation tool
	Pharmit (Sunseri and Koes, 2016)	Pharmacophore and shape-based virtual screening	Screening or optimizing TCM analogues guided by MD-derived features	Useful for ligand-based screening	Requires reliable pharmacophore or shape constraints
	Open Babel (O’Boyle et al., 2011)/OPSIN (Lowe et al., 2011)	Structure format conversion and chemical name parsing	Preparing compound files for docking and MD	Useful for batch conversion and structure generation	Complex natural products may require manual inspection
Chemical identifiers and structure tools Chemical identifiers and structure tools	UniChem (Chambers et al., 2013)/InChI (Heller et al., 2015)	Standardized chemical identifiers and cross-database mapping	Deduplication and structure consistency across databases	Useful for cross-database integration	Incorrect input structures lead to incorrect mapping
	PubChem (Kim et al., 2025)	Chemical structures, properties, synonyms, and identifiers	Obtaining and standardizing TCM constituent structures	Broad compound coverage and identifier mapping	Stereochemistry and TCM synonyms may require manual checking

## 5 Applications of molecular dynamics simulation in studies of major types of bioactive constituents in traditional Chinese medicine

### 5.1 Flavonoid constituents

Flavonoids are among the most extensively studied bioactive constituents in TCM (Wan et al., 2024). They are widely distributed in medicinal herbs such as Scutellaria baicalensis Georgi (Huangqin), Pueraria lobata (Gegen), Epimedium species (Yinyanghuo), mulberry leaves, safflower, and various TCM formulae (Wang J. et al., 2023). Representative TCM-derived flavonoids include baicalin, baicalein, wogonin, and wogonoside from Scutellaria baicalensis; puerarin and daidzein from Pueraria lobata; and icariin and icaritin from Epimedium. These compounds are commonly characterized by structural modifications such as hydroxylation, methoxylation, glycosylation, and prenylation, which may affect their hydrogen-bonding capacity, hydrophobic interactions, molecular polarity, conformational flexibility, and adaptability to target-protein binding pockets (Dias et al., 2021). Therefore, MD simulation can be used not only to validate static docking results between flavonoid constituents and their targets, but also to reveal binding stability, key interacting residues and conformational adaptation in dynamic protein environments.

Flavonoids in TCM formulae provide a research context that is more consistent with the characteristics of TCM. Taking Fufang Honghua Buji granules for the treatment of vitiligo as an example, one study combined network pharmacology, molecular docking, MD simulation, and experimental validation to identify luteolin, quercetin, and wogonin as potential key flavonoid constituents, with signal transducer and activator of transcription 3 (STAT3) as an important therapeutic target. MD simulations further showed that the complexes formed between these flavonoids and STAT3 exhibited favorable conformational stability during the simulation process. RMSD, radius of gyration, solvent-accessible surface area, hydrogen-bond number, and molecular mechanics Poisson-Boltzmann surface area (MMPBSA) binding free-energy analyses collectively supported stable interactions between these flavonoids and STAT3. This example illustrates the value of MD simulation in elucidating the mechanisms of TCM formulae (Li et al., 2023).

Puerarin is a representative isoflavone from Pueraria lobata and is also an important example in TCM flavonoid research. In an anti-influenza study, Wang et al. used molecular docking and MD simulation to analyze the interaction between puerarin and influenza virus neuraminidase. The results showed that puerarin had strong binding affinity for H1N1 neuraminidase, and MD simulation further suggested that it could maintain stable binding near the 150-loop region of neuraminidase, thereby providing a structural basis for its inhibitory effect on neuraminidase activity. This study also combined *in vitro* and *in vivo* experiments, showing that puerarin could inhibit influenza virus replication, reduce viral titers, and alleviate lung inflammation. Compared with static docking alone, MD simulation further revealed the time-dependent stability of the puerarin-binding conformation and the adaptability of the local binding pocket (Wang et al., 2021).

Icariin is a major flavonoid glycoside derived from Epimedium species. In a study of acetaminophen-induced hepatotoxicity, Shen et al. combined molecular docking, MD simulation, surface plasmon resonance, and experimental validation to investigate the interaction between icariin and S100A9. MD simulation supported a stable dynamic interaction between icariin and S100A9, while surface plasmon resonance further confirmed direct binding with an equilibrium dissociation constant of 1.14 μM. In cellular and mouse models, icariin reduced S100A9 expression and alleviated acetaminophen-induced liver injury, as indicated by decreased serum enzyme activities and hepatic apoptosis. This study demonstrates how MD simulation can help identify and characterize the direct protein targets of TCM-derived flavonoids and connect their dynamic binding behavior with experimentally observed pharmacological effects (Shen et al., 2024).

In addition to the above single-herb and formula-based examples, MD studies of flavonoids can be further used to compare how different structural modifications affect binding stability. For example, glycosylation may alter the water solubility, spatial orientation, and surface hydrogen-bond networks of flavonoid molecules, whereas methoxylation and hydroxylation may influence hydrophobic contacts, π-π stacking, and local pocket adaptability. RMSD, RMSF, hydrogen-bond occupancy, binding free-energy decomposition, and residue-level interaction analysis can help relate flavonoid structural differences to target recognition. These results may support activity comparison, quality-marker screening, and structural optimization of TCM-derived flavonoids (Dias et al., 2021).

### 5.2 Alkaloid constituents

Alkaloids are an important class of nitrogen-containing natural products in TCM and are widely distributed in medicinal herbs such as Coptis chinensis, Phellodendron amurense, Corydalis yanhusuo, Sophora flavescens, and Sinomenium acutum. Representative TCM-derived alkaloids include berberine, coptisine, and jatrorrhizine from Coptis chinensis; tetrahydropalmatine, corydaline, and protopine from Corydalis yanhusuo; matrine and oxymatrine from Sophora flavescens; and sinomenine from Sinomenium acutum (Alhas et al., 2021). Compared with flavonoids and terpenoids, alkaloids usually contain well-defined nitrogen heterocycles, exhibit stronger basicity, possess greater conformational constraints, and show distinct charge-distribution characteristics. These features allow them to form electrostatic interactions, cation-π interactions, hydrogen bonds, and hydrophobic contacts during target recognition (Kerru et al., 2020). MD simulation can further reveal the dynamic conformational stability, key residue interactions, binding free-energy contributions, and potential sources of selectivity of these alkaloids within protein-binding pockets (Honorio et al., 2021).

Isoquinoline alkaloids from Coptis chinensis are representative examples in current MD studies of TCM-derived alkaloids. In screening dipeptidyl peptidase-4 (DPP-4) inhibitors from Coptis chinensis, Zhao et al. (2024) integrated molecular docking, enzyme inhibition assays, surface plasmon resonance, and MD simulation, and found that multiple alkaloids from Coptis chinensis could directly inhibit DPP-4 activity. MD analysis showed that most DPP-4-alkaloid complexes rapidly reached equilibrium during the simulations. RMSD and RMSF analyses suggested that these alkaloids helped stabilize the DPP-4 protein conformation. MM/GBSA binding free-energy decomposition further indicated that several key residues made important contributions to binding stability. This study not only confirmed that multiple Coptis chinensis alkaloids beyond berberine also possess DPP-4 inhibitory activity, but also used MD simulation to reveal how substituents on different isoquinoline scaffolds influence target affinity and complex stability, thereby providing dynamic structural evidence for understanding the multicomponent material basis of the hypoglycemic effects of Coptis chinensis. Coptis chinensis and its related classical formulae also provide disease-oriented examples for MD-based studies of alkaloids. In a study on Coptis chinensis for chronic atrophic gastritis, Hu et al. (2024) integrated mass spectrometry, network pharmacology, molecular docking, MD simulation, and experimental validation to identify major active constituents, including berberine, coptisine, and palmatine, and investigated key targets such as PTGS2, TNF, MTOR, and TP53. MD simulation further supported berberine as one of the important active constituents responsible for the effects of Coptis chinensis, and the relevant results were further validated in cellular experiments. This study illustrates the bridging role of MD simulation in mechanistic studies of TCM alkaloids, rather than using it merely as a formal validation step after molecular docking.

Alkaloids in TCM formulae can also be investigated by MD simulation to elucidate their mechanisms of action. In a study of Yuanjiang decoction for bradyarrhythmia, Wang X. et al. (2023) adopted an integrated strategy combining liquid chromatography-mass spectrometry (LC-MS), network pharmacology, molecular docking, and MD simulation to analyze the pharmacodynamic material basis and potential targets of this formula. The results indicated that tetrahydropalmatine may be one of the key active constituents in the formula and may participate in its anti-bradyarrhythmic mechanism by interacting with cardiac ion-channel-related targets such as KCNQ1. Thus, MD simulation can contribute to mechanistic interpretation of complex relationships in TCM formulae, although the related predictions still require further functional experimental validation.

Sinomenine demonstrates the potential application of MD simulation in studying TCM alkaloids in immune- and inflammation-related diseases. A recent study combining network pharmacology, molecular docking, and MD simulation analyzed the potential mechanism of sinomenine in thyroid dysfunction and found that sinomenine and its epimer may be associated with inflammation- and immunity-related targets, including TNF, STAT3, NFKB1, IL6, SRC, ESR1, and MAPK8. Further MD simulations showed that representative complexes formed between sinomenine and ESR1 or MAPK8 exhibited favorable stability during 100 ns simulations, with RMSD, RMSF, Rg, and solvent-accessible surface area (SASA) supporting their dynamic binding stability. This study also suggests that, for TCM alkaloids with chiral centers and conformational differences, MD simulation can be used to compare the differences among different configurations or analogues in terms of target-binding stability and conformational adaptability; however, the predicted mechanisms still require further experimental validation (Chen J. et al., 2025).

Overall, MD studies of alkaloid constituents should place greater emphasis on herbal origin, structural characteristics, and disease context. For alkaloids from medicinal herbs such as Coptis chinensis, Corydalis yanhusuo, Sophora flavescens, and Sinomenium acutum, MD simulation can help explain how nitrogen heterocycles, charge distribution, methoxy substitution, chiral centers, and conformational rigidity influence target recognition, binding stability, and selectivity. Future studies should further integrate experimental affinity measurements, enzyme activity assays, cellular functional validation, and replicated MD simulations to improve the reliability, reproducibility, and biological interpretability of MD-based studies of TCM alkaloids.

### 5.3 Glycosidic constituents

Glycosidic constituents are an important class of bioactive compounds in TCM and are widely distributed in medicinal herbs such as ginseng, Astragalus membranaceus, licorice, Rehmannia glutinosa, Paeonia lactiflora, and Epimedium. According to the structural features of their aglycones, TCM-derived glycosides can be classified into several types, including saponins, flavonoid glycosides, iridoid glycosides, phenolic glycosides, and cyanogenic glycosides. Compared with most small-molecule aglycones, glycosidic constituents usually contain more hydroxyl groups, higher polarity, and greater conformational flexibility. Their sugar moieties not only affect water solubility and pharmacokinetic behavior, but may also directly participate in target recognition through hydrogen bonds, water bridges, steric effects, and membrane-interface interactions (Huang et al., 2016). Therefore, MD simulation is particularly suitable for analyzing sugar conformational changes, sugar-protein interactions, aglycone scaffold stability, and the distribution and orientation of glycosidic constituents in membrane environments (Schaub et al., 2021).

Ginsenosides are among the most representative glycosidic constituents investigated in MD studies of TCM. In a study on the protective effect of ginsenoside Rg1 against sepsis-induced acute lung injury, Zhong et al. (2024) combined network pharmacology, molecular docking, MD simulation, animal models, and *in vitro* experiments, and identified AKT1 as a potential key target responsible for the protective effect of Rg1. MD simulation further supported stable binding between Rg1 and AKT1. Experimental results showed that Rg1 alleviated sepsis-related lung injury, reduced inflammatory cytokine levels, and inhibited apoptosis in lung epithelial cells. This study illustrates the bridging role of MD simulation in mechanistic studies of TCM-derived glycosidic constituents.

In addition to target-protein binding, interactions between glycosidic constituents and membrane environments represent another important application scenario for MD simulation. Ginsenoside Rh2 consists of a triterpenoid sapogenin and a glucose moiety, giving it both a hydrophobic scaffold and a hydrophilic sugar group and therefore clear amphiphilic properties. Membrane simulation studies have shown that the orientation and distribution of Rh2 in lipid membranes are closely related to its sapogenin scaffold, sugar position, and membrane lipid composition. MD simulation can dynamically track the insertion, orientational adjustment, hydrogen-bond formation, and local lipid perturbation of Rh2 at the membrane interface, thereby explaining how the sugar moiety contributes to membrane-surface positioning and how the aglycone portion interacts with lipid hydrophobic tails (Garza-Miyazato et al., 2024). Similar studies are important for understanding the membrane activity, cellular permeability, and potential cytotoxic mechanisms of ginsenosides.

In a study on astragaloside IV for idiopathic pulmonary fibrosis, Chen et al. (2025b) integrated transcriptomic data, network pharmacology, molecular docking, MD simulation, and *in vivo* and *in vitro* experiments, and identified phosphatidylinositol-4,5-bisphosphate 3-kinase catalytic subunit alpha (PIK3CA) as a potential key target through which astragaloside IV regulates the PI3K-AKT pathway. MD simulation showed that the astragaloside IV-PIK3CA complex maintained favorable stability during the simulation. RMSD, RMSF, Rg, SASA, hydrogen-bond number, and MM-GBSA results collectively supported its binding stability, with residues such as ILE932 and GLU849 contributing substantially to the binding free energy. Further cellular and animal experiments showed that astragaloside IV inhibited fibroblast activation and alleviated pulmonary fibrosis. This example indicates that MD simulation can not only describe the binding mode between glycosidic constituents and targets, but also be integrated with disease-model experiments to explain pathway-regulatory mechanisms of TCM-derived saponins.

For TCM-derived glycosidic constituents, the value of MD simulation lies not only in validating molecular docking results, but also in revealing the cooperative roles of sugar moieties and aglycone scaffolds. For example, sugar moieties may influence target recognition through hydrogen bonds and water bridges, whereas aglycone scaffolds may maintain binding stability through hydrophobic interactions and steric complementarity. Amphiphilic glycosides may also affect cellular uptake and pharmacological effects through their distribution at membrane interfaces. Future studies should pay greater attention to how the number of sugar moieties, glycosidic linkage patterns, aglycone configurations, and membrane environments influence binding stability and pharmacodynamic differences. Replicated MD simulations, enhanced sampling, free-energy calculations, and experimental validation should be further integrated to establish a dynamic structure-activity framework more suitable for TCM-derived glycosidic constituents.

### 5.4 Terpenoid constituents

Terpenoids are an important class of natural products in TCM. They are generally composed of isoprene units and can be classified into monoterpenoids, sesquiterpenoids, diterpenoids, and triterpenoids. Representative TCM-derived terpenoids include artemisinin from Artemisia annua, tanshinone IIA from Salvia miltiorrhiza, andrographolide from Andrographis paniculata, and various monoterpenoid and sesquiterpenoid constituents from volatile-oil-containing medicinal herbs (Masyita et al., 2022). Compared with flavonoids and alkaloids, terpenoids usually exhibit stronger hydrophobicity, complex cyclic scaffolds, multiple chiral centers, and high conformational specificity (Hosseini and Pereira, 2023). Their target-recognition processes therefore often involve hydrophobic-pocket accommodation, induced fit, membrane-environment distribution, and local conformational modulation. MD simulation can be used to analyze orientational changes of terpenoids in hydrophobic pockets, binding-conformation stability, contributions of key hydrophobic residues, and the effects of membrane environments on their distribution and target accessibility (Raza et al., 2023).

Artemisinin and its derivatives are representative sesquiterpene lactones derived from Artemisia annua, among which dihydroartemisinin is an important active derivative of artemisinin. In a study investigating the anti-esophageal cancer mechanism of dihydroartemisinin, Wang (2022) combined network pharmacology, molecular docking, and MD simulation to systematically analyze the potential interactions between dihydroartemisinin and esophageal cancer-related targets. The study first predicted candidate targets of dihydroartemisinin and then integrated differentially expressed genes in esophageal cancer to identify key targets such as ADORA2B and AURKA. Molecular docking was subsequently used to analyze the binding modes of dihydroartemisinin with these candidate targets, and MD simulation was further applied to evaluate the dynamic stability of the dihydroartemisinin-ADORA2B and dihydroartemisinin-AURKA complexes. The results showed that dihydroartemisinin could form relatively stable binding states with these targets, and MD trajectories further supported the reliability of the docking conformations. For artemisinin-type TCM-derived terpenoids, MD simulation can not only validate static docking results, but also transform multitarget relationships predicted by network pharmacology into molecular mechanistic hypotheses supported by dynamic structural evidence.

In addition to artemisinin, tanshinone constituents from Salvia miltiorrhiza provide another TCM-relevant example for MD studies of terpenoids. Tanshinone IIA is a lipophilic diterpene quinone from Salvia miltiorrhiza and has multiple pharmacological activities, including anti-inflammatory, cardiovascular-protective, and antitumor effects. In a study investigating the mechanism of tanshinone IIA against prostate cancer, Zhang T. et al. (2023) combined transcriptomic bioinformatics, molecular docking, and MD simulation, and identified PPARG as a potential target of tanshinone IIA. MD simulation was further used to evaluate the dynamic stability of the tanshinone IIA-PPARG complex, supporting the plausibility of this candidate target from the perspectives of conformational fluctuation, binding stability, and key molecular interactions. This study indicates that, for hydrophobic TCM-derived terpenoids such as tanshinones, MD simulation can not only validate docking poses, but also help explain their conformational adaptation and stable binding mechanisms within hydrophobic protein pockets.

Andrographolide is a representative diterpene lactone from Andrographis paniculata and is also an important example in MD studies of TCM-derived terpenoids. In a study of non-small-cell lung cancer, Wang et al. (2022) found that andrographolide suppressed tumor progression by regulating autophagy and antitumor immune responses. Further analysis showed that andrographolide inhibited the JAK2/STAT3 signaling pathway, promoted autophagic degradation of PD-L1, and enhanced the efficacy of anti-PD-1 immunotherapy. MD simulation indicated that andrographolide could directly bind to STAT3 with high affinity, providing a structural basis for its regulation of STAT3-related signaling. This study illustrates the role of MD simulation in linking TCM-derived terpenoid constituents, key target engagement, immune-regulatory pathways, and experimental pharmacological phenotypes. It also suggests that MD studies of TCM-derived terpenoids should move beyond simple binding-stability validation toward disease-contextualized and pathway-oriented mechanistic interpretation.

Overall, MD studies of TCM-derived terpenoids should focus on both their structural features and TCM-related application contexts. For artemisinin, tanshinone IIA, andrographolide, and related terpenoids, MD should not be limited to validating ligand-target complex stability. It can also help explain how hydrophobic scaffolds, lactone rings, quinone moieties, peroxide bridges, and chiral centers influence target recognition, structural adaptation, membrane distribution, and downstream pathway regulation.

### 5.5 Polysaccharide constituents

Polysaccharides are important macromolecular bioactive substances in TCM and are widely distributed in medicinal herbs such as Ganoderma lucidum, Astragalus membranaceus, Lycium barbarum, Lentinula edodes, Dendrobium officinale, Poria cocos, and Angelica sinensis. Unlike small molecules such as flavonoids, alkaloids, and terpenoids, TCM polysaccharides usually do not exert their effects through a single high-affinity binding site. Instead, their biological activities are more often associated with structural features such as molecular weight, monosaccharide composition, glycosidic linkage patterns, branching architecture, triple-helix conformation, and chain flexibility (Ferreira et al., 2015). These features contribute to multivalent weak interactions, pattern-recognition receptor binding, immunoregulation, and intestinal barrier modulation. Therefore, the value of MD simulation in polysaccharide research should not be understood merely as post-docking stability validation, but rather as a means to explain how polysaccharide chain conformations, receptor-recognition patterns, hydration layers, chain flexibility, and multivalent interactions influence biological activity (Nieto-Fabregat et al., 2024).

The interaction between lentinan and the Dectin-1 receptor is a representative example of MD studies on polysaccharides. Lentinan is a β-1,3-D-glucan with well-established immunostimulatory activity. Wu et al. (2021) used MD simulation to analyze the higher-order structure of lentinan and its interaction with the innate immune receptor Dectin-1. The results showed that lentinan could form a curved triple-helical conformation in aqueous solution, with flexible side chains, and could participate in Dectin-1 recognition through hydrogen bonds, CH-π interactions, and multivalent weak interactions. Binding of lentinan may induce conformational changes in local domains of Dectin-1, thereby providing a molecular-level explanation for its immunostimulatory effect. MD simulation can reveal dynamic polysaccharide-chain conformations and receptor-recognition processes that are difficult to capture using conventional structural characterization methods, thus providing a basis for interpreting the immunomodulatory mechanisms of polysaccharide constituents.

Lycium barbarum polysaccharides further illustrate the value of MD simulation in elucidating the relationship among “structural parameters-receptor affinity-immune effects.” These polysaccharides exhibit various biological activities, including immunoregulation, antioxidant effects, and intestinal barrier protection, which are closely related to molecular-weight distribution, monosaccharide composition, and glycan-chain conformation. Guo et al. (2024) investigated the effects of molecular weight and gastrointestinal digestion on the immunoregulatory activity of Lycium barbarum polysaccharides, with particular focus on their interaction with the Toll-like receptor 4/MD-2 receptor complex. The results showed that polysaccharide fractions with different molecular weights differed in their binding ability to TLR4/MD-2 and in their immune-inducing effects, with medium-molecular-weight fractions generally exhibiting stronger receptor-binding capacity and cytokine-inducing activity. MD and related interaction analyses further explained how monosaccharide components such as arabinose and galactose, as well as glycan-chain spatial conformations, affect receptor recognition. Such studies link molecular weight, monosaccharide composition, and receptor-binding behavior, helping to explain the sources of activity differences among TCM polysaccharides from a dynamic structural perspective.

Dendrobium officinale polysaccharides have been reported to regulate intestinal immunity, improve intestinal mucosal barrier function, and exert anti-inflammatory effects. Yang et al. (2024) integrated network pharmacology, molecular docking, MD simulation, and *in vivo* experiments, and identified TLR4 as a potential key target involved in the intestinal immunomodulatory effects of Dendrobium officinale polysaccharides. Molecular docking and MD simulation further supported favorable affinity and complex stability between Dendrobium officinale polysaccharides and TLR4. Animal experiments also showed that these polysaccharides could regulate the expression of TLR4 and its downstream related proteins in colonic mucosa. This study illustrates the bridging role of MD simulation in mechanistic studies of TCM polysaccharides.

Because TCM polysaccharides usually have high molecular weights, complex glycosidic linkage patterns, substantial chain flexibility, and extensive hydration layers, all-atom MD alone is often insufficient to fully capture their long-timescale conformational changes, aggregation behavior, and multivalent receptor-recognition processes. Coarse-grained MD provides an important complementary approach to address this limitation. By reducing the number of degrees of freedom, coarse-grained MD extends the accessible spatial and temporal scales and is suitable for investigating polysaccharide chain folding, aggregation, self-assembly, membrane-surface distribution, and multivalent interactions with pattern-recognition receptors. The Martini force field is one of the most widely used coarse-grained force fields; both its early versions and Martini 3 have been applied to coarse-grained simulations of various biomolecular systems (Marrink et al., 2007; Souza et al., 2021). In recent years, Martini 3 has also been extended to carbohydrate systems, providing a parameterization basis for coarse-grained simulations of polysaccharides, glycolipids, and glycan-related complexes (Grünewald et al., 2022). Therefore, coarse-grained models such as Martini may serve as useful tools for simulating polysaccharide-lipid membrane-receptor complex systems and for analyzing polysaccharide-induced receptor clustering, membrane-surface enrichment, and large-scale supramolecular assembly processes. Mesoscale modeling may further extend this framework by describing polysaccharide network formation, gel-like assemblies, nanoparticle delivery systems, and receptor-clustering behavior at spatial and temporal scales that are difficult to access using all-atom or conventional CG-MD alone.

## 6 Limitations and challenges

Taken together, current studies suggest that MD simulation provides a distinctive dynamic perspective for investigating representative compound-target interactions in TCM research. Rather than fully reproducing formula-level multicomponent synergy, most current MD studies provide dynamic molecular evidence for selected key interactions and support the gradual development of more mechanism-oriented TCM research.

### 6.1 Identification and selection of active constituents

In the application of MD simulation to TCM research, the identification and selection of active constituents is one of the key steps. TCM prescriptions contain not only pharmacologically active compounds, but also metabolites, excipients, impurities, and even potentially toxic components, which makes the direct selection of candidate compounds for MD simulation inherently uncertain and, at times, subjective (Cheng et al., 2023).

At present, component screening mainly relies on physicochemical criteria provided by TCM databases, such as oral bioavailability (OB) and drug-likeness (DL), but these static parameters do not necessarily reflect *in vivo* activity accurately (Wang et al., 2024). Most databases do not comprehensively cover active metabolites formed after *in vivo* biotransformation of TCM constituents, which may lead to the exclusion of substances that truly interact with the target. At the same time, some highly active trace constituents may be excluded from the simulation system because of low abundance or missing data. In MD practice, the accuracy and representativeness of the input chemical structures directly affect the biological interpretability of the simulation results. For example, if a constituent has already undergone structural transformation *in vivo*, such as glycosidic hydrolysis or hydroxylation, but the original structure is still used for simulation, the simulated molecule may deviate from the actual pharmacologically relevant species (Xue et al., 2025).

The overall efficacy of TCM arises from the synergistic actions of multiple constituents under the compatibility principle of sovereign, minister, assistant, and courier (Li et al., 2022). However, due to computational limitations and the finite accuracy of force fields, most current MD simulation studies are still restricted to simplified single-component-single-target models. More realistic multicomponent and multitarget models remain difficult to construct. Key challenges include uncertain component ratios, overlapping or allosterically coupled binding sites, and rapidly increasing computational complexity. These factors make it extremely difficult to systematically elucidate TCM compatibility rules at the molecular level (Li Y. et al., 2025).

### 6.2 Representation of dose-effect relationships

MD simulation has clear strengths in qualitatively explaining the binding modes between active TCM constituents and their targets, but it shows obvious limitations when addressing the core pharmacodynamic issue of dose-response relationships (Redelmeier and Zipursky, 2023). Changes in TCM dosage affect not only efficacy but also toxicity. If dose-effect relationships are ignored and predictions rely solely on single-conformation analysis and binding-energy evaluation, the result may be one-sided efficacy prediction and biased risk assessment.

Dose-effect relationships *in vivo*, such as metabolic rate, tissue distribution, and duration of peak plasma concentration, are jointly regulated by multiple factors (Ma et al., 2024), and pharmacokinetic behavior cannot be directly captured by MD simulation. For example, some constituents may show beneficial effects at low doses, but at high doses may cause toxicity or antagonistic effects because of binding-site saturation or the activation of off-target pathways. Such effects are difficult to identify through structure-based simulation alone. Some studies have attempted to correlate MD simulation results with dose-response parameters such as IC50 and Ki by establishing fitted models to predict the functional effect associated with a given conformation (Roadnight Sheehan et al., 2024). However, because simulation times are inherently limited, usually to the scale of hundreds of nanoseconds or microseconds, they are insufficient to cover the full ensemble of conformational changes brought about by dose modulation, and therefore lack systematicity and quantitative comparability (Kordylewski et al., 2025).

Thus, the limited ability of current MD approaches to represent dose–effect relationships remains a major obstacle in TCM mechanism research. New multiscale methods and dynamic concentration models are needed to better reflect the behavior of TCM systems *in vivo*.

### 6.3 Limitations of hardware and software technologies

Although MD simulation has gradually become one of the mainstream computational tools for revealing interactions between TCM constituents and their targets, its practical application is still constrained by current computational resources, simulation algorithms, and the level of data standardization.

At the hardware level, high-precision, all-atom simulations on long timescales still require substantial computational power (Soares et al., 2023). Because TCM formulae involve multiple constituents and multiple targets, the size of the simulation system increases markedly if one aims to build a model that realistically reflects multicomponent synergy. Even with GPU-accelerated platforms, microsecond-level simulations or repeated trajectories still require considerable time and substantial resource investment (Ojha et al., 2024). As a result, limited computing allocation and hardware availability mean that some studies remain confined to relatively short timescales or simplified systems, and are therefore unable to fully capture the slow conformational changes of complex systems (Ahmed et al., 2023).

At the level of software algorithms, MD platforms also show limitations when dealing with constituent types that are characteristic of TCM. For example, some TCM components, such as glycosides, phenolics, and polysaccharides, have complex spatial structures and unusual polarity distributions. Standard force fields often lack specific parameters for such molecules, which reduces simulation accuracy (López et al., 2009). Although this can in principle be addressed by manual parameterization, the process is time-consuming, labor-intensive, and requires a high level of technical expertise from the user (Kognole et al., 2022). In addition to these parameterization challenges, all-atom MD is also constrained by the high computational cost of modeling large, multicomponent, and long-timescale TCM systems (Liwo et al., 2021). In this regard, coarse-grained molecular dynamics (CG-MD) provides a useful complementary strategy. By reducing the number of degrees of freedom, CG-MD can extend the accessible spatial and temporal scales and is therefore suitable for studying multicomponent coexistence, membrane distribution, molecular aggregation, and long-range conformational changes in complex TCM systems. However, the application of CG-MD to TCM research remains limited by reduced structural resolution and insufficient coarse-grained parameters for many structurally diverse natural products. Therefore, future studies should combine CG-MD with all-atom MD and experimental validation to construct more reliable multiscale simulation workflows.

### 6.4 Diversity of targets and their activities

In TCM research, target identification often depends on indirect prediction methods such as network pharmacology and gene enrichment analysis, and is not always based on clear experimental validation (Ren et al., 2023). These predicted targets often involve structural uncertainty. High-resolution structures may be unavailable, and available structures often represent only one conformational state. Under real physiological conditions, however, proteins commonly exist in multiple conformations and undergo state transitions, which can introduce bias into model construction (Guo et al., 2023).

Many TCM targets are highly dynamic and structurally variable proteins, such as membrane proteins, receptor kinases, and nuclear transcription factors (Salas-Estrada et al., 2022). For example, the active conformations of G protein-coupled receptors (GPCRs) involve coordinated motions among multiple transmembrane helices (Zhang et al., 2024). Yet many molecular simulations still rely on docking strategies that treat the target as rigid and the ligand as flexible, which cannot reproduce dynamic binding pathways or induced-fit processes and therefore limits the biological interpretability of the simulation.

The functional diversity of TCM targets further complicates simulation-based evaluation. As noted by Ryszkiewicz et al. (2025), multi-target-directed ligands (MTDLs) can regulate multiple biological targets simultaneously, and even if one target is identified, its activity state and conformation may still vary. Xie et al. (2023) further pointed out that a large number of protein structures and states remain inadequately resolved. A single constituent may act on multiple targets and exert entirely different effects in different tissues or organs; even the same target may produce promoting or antagonistic effects under different activation states. Certain kinase proteins, for example, may exhibit activation when bound to a TCM constituent in one cellular context, but inhibition in another. Such bidirectional regulation is difficult to reveal through MD simulation based on a single structural conformation (Lembo and Bottegoni, 2024).

Experimental validation is essential for determining whether MD-derived predictions are biologically meaningful. Biophysical assays, such as surface plasmon resonance, isothermal titration calorimetry, microscale thermophoresis, bio-layer interferometry, differential scanning fluorimetry, and cellular thermal shift assay, can be used to verify ligand-target binding, binding affinity, and target engagement (Sanchez et al., 2022). Site-directed mutagenesis and competitive binding assays can test whether residues or binding pockets predicted by MD are functionally important. Structural methods, including cryo-electron microscopy, X-ray crystallography, and nuclear magnetic resonance, can provide evidence for ligand-bound conformations, allosteric states, and receptor-ligand recognition modes (García-Nafría and Tate, 2021). For dynamic processes that are difficult to capture using static structures, nuclear magnetic resonance (NMR) relaxation experiments, hydrogen-deuterium exchange mass spectrometry, and fluorescence resonance energy transfer can help examine conformational fluctuations and allosteric communication. In TCM research, such validation is particularly important because predicted compound–target interactions must be interpreted in the context of complex constituents, metabolic transformation, dose exposure, and pathway-level biological effects (Jiashuo et al., 2022).

## 7 Summary

MD simulation has become an important tool for TCM mechanism research because it can provide time-resolved molecular evidence for ligand-target recognition, structural adaptation, hidden binding sites, membrane-associated behavior, and binding energetics. Compared with static docking or correlation-based network analysis, MD helps transform predicted compound-target associations into mechanistic hypotheses that can be further examined experimentally.

However, the application of MD simulation in TCM remains, overall, at an early stage of transition from descriptive validation to mechanism-oriented prediction. Most studies still follow simplified representative compound-target models. These studies are useful for validating key interactions predicted by network pharmacology or molecular docking, but they should not be regarded as complete simulations of formula-level multicomponent synergy. Their main value lies in providing dynamic molecular evidence for selected nodes or interactions within broader compound-target-pathway networks. Dynamic network models that systematically capture synergistic and antagonistic interactions among multiple constituents in TCM formulae have not yet been fully established. In addition, the biological interpretation of simulation results is still often limited to surface-level parameters such as binding free energy and conformational stability, and there remains a lack of multiscale, multidimensional integrative analytical frameworks that truly match the holistic and dynamic perspectives of TCM. In view of the major challenges posed by the ambiguous definition of active constituents, the difficulty of representing dose-effect relationships, the high dynamism of complex targets such as membrane proteins, and limitations in computational resources and force-field accuracy, methodological and application-level innovation is urgently needed.

Future MD-based TCM research should move beyond post-docking stability validation toward integrated, multiscale, and mechanism-oriented analysis. When combined with TCM databases, network pharmacology, multi-omics evidence, and AI-assisted modeling, MD can provide dynamic molecular evidence for target engagement, conformational adaptation, membrane-associated behavior, and potential competitive or cooperative effects among representative constituents. Thus, MD can serve as a key methodological bridge between static compound-target predictions and the complex biological effects of TCM, offering molecular-level support for TCM modernization and mechanism-driven development.
